# Prevalence, incidence, and distribution of human papillomavirus types in female sex workers in Kenya

**DOI:** 10.1177/0956462419884454

**Published:** 2020-01-16

**Authors:** Kristen Sweet, Claire Bosire, Busola Sanusi, Carly J Sherrod, Jessie Kwatampora, Wairimu Waweru, Nelly Mugo, Joshua Kimani, Jie Ting, Jennifer Clark, Dirk P Dittmer, Jennifer S Smith

**Affiliations:** 1 Curriculum in Genetics and Molecular Biology, School of Medicine, University of North Carolina, Chapel Hill, NC, USA; 2 Department of Health Behavior, University of North Carolina, Chapel Hill, NC, USA; 3 Department of Biostatistics, University of North Carolina, Chapel Hill, NC, USA; 4 Lineberger Comprehensive Cancer Center, University of North Carolina, Chapel Hill, NC, USA; 5 Curriculum in Health Policy and Management, Gilling’s School of Global Public Health, University of North Carolina, Chapel Hill, NC, USA; 6 Institute of Tropical and Infectious Diseases, University of Nairobi, Nairobi, Kenya; 7 Department of Pathology, College of Health Sciences, University of Nairobi, Nairobi, Kenya; 8 Kenya Medical Research Institute, Nairobi, Kenya; 9 Department of Epidemiology, University of North Carolina, Chapel Hill, NC, USA; 10 Department of Microbiology and Immunology, School of Medicine, University of North Carolina, Chapel Hill, NC, USA

**Keywords:** Human papillomavirus, HIV, cervical dysplasia, sex workers, Africa

## Abstract

Female sex workers (FSWs) have a notably high risk of acquiring human papillomavirus (HPV) infections. Relatively few studies address the type-specific prevalence and incidence of HPV among FSWs in sub-Saharan Africa. FSWs (n = 348) attending the Korogocho clinic in Nairobi, Kenya participated from August 2009 to March 2011. HPV DNA was detected using the SPF10-LiPA25 PCR assay. Baseline prevalence of HPV infection and cervical dysplasia were calculated, stratified by HIV-serostatus. Incidence rate (IR) of infection was calculated as number of new infections from baseline over person-months among 160 HPV-negative participants with complete 12-month follow-up. Baseline HPV prevalence was 23.6% for any HPV and 20.4% for high-risk HPV (hrHPV) types. Most prevalent types were HPV52 (10.1%), HPV35 (2.3%), and HPV51 (2.3%). A quarter (24%) of participants were HIV-positive. HPV prevalence was higher in HIV-positive (32.1%) than HIV-negative (20.8%) participants. hrHPV prevalence was higher in HIV-positive (27.4%) than HIV-negative (18.2%) women. During follow-up, HPV IR was 31.4 (95% CI: 23.8–41.5) for any HPV and 24.2 (95% CI: 17.9–32.8) for hrHPV types. HPV52 had the highest IR (6.0; 95% CI: 6.5–10.3). Overall HPV and hrHPV prevalence were lower than expected, but both prevalence and incidence were higher in HIV-positive than in HIV-negative women.

## Introduction

Invasive cervical cancer (ICC) is the leading cause of cancer among women in Eastern Africa.^1^ More than 85% of the estimated 311,365 ICC deaths in 2018 occurred in low- and middle-income countries, where screening remains inadequate.^2^ Nearly 100% of ICC are caused by high-risk (oncogenic) human papillomavirus (HPV) infections.^3^ HIV-positive women have a significantly higher risk of HPV infection overall, multiple types of HPV infections, and HPV persistence than non-infected women.^4,5^ HIV-positive women also have higher rates of high-grade cervical precancer and ICC than HIV-negative women.^6,7^

HPV types can be separated into two categories: high-risk types (high-risk HPV [hrHPV]) and low-risk types (low-risk HPV [lrHPV]), based on their association with ICC.^8^ Primary prevention of HPV infections through vaccination could reduce the burden of high-risk type HPV infections and associated cervical disease. There are currently three FDA-approved vaccines: the bivalent vaccine which protects against hrHPV types 16 and 18; the quadrivalent vaccine which protects against hrHPV types 16 and 18^9,10^ and low-risk HPV types 6 and 11 (which cause genital warts); and the most recent nonavalent vaccine, which protects against 6, 11, 16, 18 and an additional five hrHPV types (31, 33, 45, 52, and 58).^11^

An increasing number of studies have reported on the type-specific prevalence of HPV among women in sub-Saharan Africa,^12–16^ although longitudinal studies on HPV incidence, particularly among populations at increased risk of acquiring sexually transmitted infections (STIs) are still scarce.^17–19^

In this study, we sought to characterize the baseline prevalence, incidence, and genotype distribution of HPV infection in 348 female sex workers (FSWs) from the Korogocho neighborhood in Nairobi, Kenya using a highly sensitive, type-specific DNA assay. Additionally, we examined the burden of HPV and HPV-associated cervical disease, stratified by HIV-serostatus, to better understand differences in disease burden in HIV-positive as compared to HIV-negative women.

## Materials and methods

### Study population

The Korogocho clinic in Nairobi, Kenya, provides counseling and medical care, including screening and treatment of cervical cancer and STIs for women currently working as FSWs in the Korogocho area. The study population was selected among FSWs attending the clinic from August 2009 to March 2011. Women were eligible to participate if they were 18–50 years of age and provided informed consent which covered the study objectives, details of the study procedures, and the plans for storage and transportation of samples. To account for a population of low literacy, questionnaires were verbally administered by a trained nurse with extensive interview experience. Women were not eligible for the study if they had undergone a hysterectomy or were in the second or third trimester of pregnancy. At a study-associated clinic, visits were conducted in three-month intervals. Specimens for HPV testing were collected every three months and cervical specimens for cytology examinations were collected every six months. We considered months 0, 6, and 12 for our analyses.

Ethical approval for the study was granted by the Institutional Review Boards of Kenyatta National Hospital, Nairobi, Kenya and the University of North Carolina at Chapel Hill, USA. The study was conducted in accordance with all applicable ethical standards for research on human subjects.

### Sample collection and laboratory analyses

Sociodemographic, reproductive, and sexual behavior information was collected at baseline via a structured questionnaire. Each participant underwent a pelvic examination where the physician collected cervical specimens for HPV DNA testing, cytology, and histology. Blood samples were collected for HIV testing. Specific details on the collection of cervical specimens for HPV DNA testing and conventional Papanicolaou testing have been described in detail elsewhere.^20^

Pap smears were evaluated at the University of Nairobi and classified according to the 2001 Bethesda System for reporting cervical cytology.^21^ All smears were independently graded by two expert cytopathologists who were blinded to the HPV and other laboratory results; the final cytological diagnosis was based on their consensus. Women with low-grade squamous intraepithelial lesions (LSILs) or atypical squamous cells of undetermined significance (ASCUS) were instructed to undergo a repeat cytology four months later. Women with high-grade squamous intraepithelial lesions (HSILs) or ASCUS with possibility of high-grade (ASCUS-H) changes were immediately referred to a colposcopy-directed biopsy for histologic confirmation. Women diagnosed with histological cervical intraepithelial neoplasia 2 or worse (CIN2+) received standard care and treatment at the Kenyatta National Hospital.

The samples were also tested for *Chlamydia trachomatis* (CT), *Neisseria gonorrhoeae* (GC), *Trichomonas vaginalis* (TV), and *Mycoplasma genitalium* (MG) using the Aptima STI assay (Hologic Corporation, San Diego, CA). Women with a positive result for a treatable STI based on laboratory diagnoses were immediately recalled and treated. Women with symptomatic STIs were treated at the same clinical visit based on syndromic management. Based on the Kenyan Ministry of Health provision on management of reproductive tract infections, syndromic management was used after the assessment of vaginal discharge.^22^

Participant serum was tested for HIV antibodies by enzyme-linked immunosorbent assay (ELISA) using the Detect HIV-1 kit (BioChem ImmunoSystems Inc., Montreal, Canada), and positive results were confirmed by a second ELISA. Peripheral blood CD4^+^ T cells were also ascertained. HIV ELISA and CD4 assay testing were conducted at the University of Nairobi. All assays were performed according to the manufacturers’ instructions, with technicians blinded to the cytology, laboratory, and other study results.

### HPV detection and typing

HPV DNA detection was performed at the University of North Carolina (Chapel Hill, NC) using the HPV SPF10-LiPA25 test, an in vitro PCR DNA ELISA (PCR/DEIA) for qualitative and highly sensitive detection of HPV (Labo Biomedical Products, DDL Diagnostic Laboratory, Voorburg, Netherlands).^23^ Briefly, DNA was extracted from 1 ml of ThinPrep solution using a MagnaPure96 robotic workstation (Roche Diagnostics, Indianapolis, IN). For the DEIA assay, DNA was amplified using biotinylated PCR primers designed to amplify 44 anogenital HPV types. Biotinylated products were hybridized to streptavidin-coated wells, which were hybridized to conjugated HPV-specific probes. Samples were analyzed by an ELISA plate reader and scored as HPV positive, negative, or borderline by comparing their optical density value to that of a standard. Since the DEIA assay can recognize 44 known HPV types but does not provide type information, it was used as an initial screening step to determine HPV positivity.

To determine specific HPV types, biotinylated PCR products for samples that scored positive or borderline on the DEIA assay were analyzed using a reverse hybridization assay kit SPF10-LiPA25 (Labo Biomedical Products, DDL Diagnostic Laboratory, Voorburg, Netherlands). Denatured amplicons were hybridized to membrane strips coated with type-specific oligonucleotide probes, and streptavidin-conjugated alkaline phosphatase was added. Positive samples were visualized by adding BCIP/NBT chromogen to the membranes. A purple band indicated positivity, and the band position on the membrane was used to determine the HPV type(s). The 25 HPV types that could be identified by this assay include 6, 11, 16, 18, 31, 33, 34, 35, 39, 40, 42, 43, 44, 45, 51, 52, 53, 54, 56, 58, 59, 66, 68/73, 70, and 74. The sequence variation within the SPF10-LiPA25 primers allows the recognition of these different HPV types, except for 68 and 73, as their inner primer regions are identical and cannot be distinguished from each other in the test.

Samples that scored positive by the DEIA assay but negative by the SPF10-LiPA25 assay were defined as ‘Type X’ (n = 18; 3, 4, 5, 7, 8, 13, 26, 27, 30, 32, 37, 55, 61, 62, 64, 65, 67, 69, or 71). Consensus MY09/MY11 polymerase chain reaction (PCR) assay was used to analyze their HPV positivity. Amplification was performed with 2X GoTaq Green Master Mix (Promega, cat# M7122) with the following cycling conditions: 95°C for 5 min followed by 40 cycles of 1 min denaturation at 95°C, 1 min annealing at 50°C, and 1 min elongation at 72°C. The final cycle was followed by a final elongation step at 72°C for 5 min. Positive and negative control samples were included for each PCR experiment. PCR products were analyzed on a 1.2% agarose gel prepared with 1X modified TAE (pH = 8.0), stained with ethidium bromide and visualized by UV transillumination. HPV positive samples were excised from the gel, purified using the Ultrafree DA kit (EMD Millipore, cat.# 42600) and ligated into a pGEM-T vector using the pGEM-T Easy Vector System I (Promega, cat.# A1360). Individual clones were sent for sequence validation (Genewiz Inc., San Diego, California). The sequences were subjected to a BLAST search and further analyzed by using CLC Genomics Workbench software (CLC Bio, version 7.0.4). HPV31 was used as a reference. HPV results were used for research purposes only.

### Statistical analyses

We calculated the prevalence of HPV DNA infection and cervical dysplasia at baseline, stratified by participant HIV serology status. HPV status was classified as follows: any HPV (positive for at least one HPV type); hrHPV (16, 18, 31, 33, 35, 39, 45, 51, 52, 56, 58, 66, 68/73); or lrHPV (6, 11, 44, 53, 54, 70, 74). While HPV73 is not a high-risk type, differentiation between 73 and 68 was not possible with the SPF10-LiPA25 assay. To minimize the type II error rate for hrHPV status, samples that contained HPV68/73 were considered high-risk. Women with multiple types were considered hrHPV if at least one high-risk type was detected. Women with uncategorized HPV type were excluded from our analysis of hrHPV or lrHPV type groupings. The Fisher’s exact test was performed to compare the baseline prevalence of HPV, cytology, and cervical dysplasia among HIV-positive versus HIV-negative FSWs. Odds ratios and corresponding 95% confidence intervals were calculated to examine the relationship between hrHPV and baseline characteristics.

For the longitudinal analyses, 141 women who had one or more missing samples during the follow-up visits were excluded, resulting in a sample size of 207 FSWs with data collected at all three time points: baseline, 6 months, and 12 months. Incidence rates (IRs) for HPV detection over the 12-month period for the population at risk were estimated for individual HPV types and for specific HPV groupings: any HPV, hrHPV, or lrHPV. An incident HPV infection was defined as the presence of a specific type of HPV not detected at the time point immediately prior (i.e. baseline for 6 months or 6 months for 12 months). The IR of infection was calculated as the number of new infections from baseline over the number of person-months. The detection of multiple novel types of HPV in a single visit was considered to have occurred from a single acquisition event. For our analyses, p < 0.05 was considered significant. All statistical analyses were conducted using SAS version 9.4 (SAS Institute, Cary, NC, USA).

## Results

### Baseline characteristics

The median age of the 348 women participating in this study was 28 years (range, 18–48; Table 1). The median duration of sex work was 16 years (range, 10–25), with a median of 10 (range, 2–40) sexual clients per week (Table 1). At the baseline, approximately a quarter of the women (n = 84; 24.1%) tested positive for HIV. The prevalence of CT was 3.8% (13/345), GC 2.3% (8/346), TV 7.2% (25/346), and MG 13.0% (45/346). The prevalence of individual STIs in HIV-positive women was similar as in HIV-negative women except for CT which was higher in HIV-negative women (4.9%) compared to HIV-positive (0%, p = 0.04).

**Table 1.** Prevalence of high-risk (hr) HPV infection among 348 female sex workers in Nairobi, Kenya, stratified by HIV status.

	Overall (n = 348)	HIV-positive (n = 84)			HIV-negative (n = 264)		
Characteristic	N (%)	n	hrHPV positiven (%)	OR (95% CI)	n	hrHPV positiven (%)	OR (95% CI)
Age, median (range), years	28 (18–48)		32 (21–48)				27 (18–46)
≥30	197 (62.1)	57	13 (18.0)	1.0	94	12 (12.8)	1.0
<30	151 (37.9)	27	10 (41.2)	2.0 (0.7, 5.4)	170	36 (21.2)	1.8 (0.9, 3.7)
Education, years^a^							
≤8	264 (75.9)	65	18 (27.7)	1.0	199	33 (16.6)	1.0
>8	82 (23.6)	18	5 (27.8)	1.0 (0.3, 3.2)	64	15 (23.4)	1.5 (0.8, 3.1)
Marital status							
Single/never married	155 (44.5)	23	9 (39.1)	1.0	132	30 (22.7)	1.0
Married/Co-habiting	3 (0.9)	1	0 (0.0)	–	2	0 (0.0)	–
Divorced/Widowed/Separated	190 (54.6)	60	14 (23.3)	0.5 (0.2, 1.3)	130	18 (13.9)	0.5 (0.3, 1.0)
Age at first sexual intercourse, years							
≥16	214 (61.5)	40	13 (32.5)	1.0	174	34 (19.5)	1.0
<16	134 (38.5)	44	10 (22.7)	0.6 (0.2, 1.6)	90	14 (15.7)	0.8 (0.4, 1.5)
Sexual clients/week, median (range)	10 (2–40)		11 (3–40)			10 (2–40)	
≤10	195	42	15 (35.7)	1.0	153	27 (17.6)	1.0
>10	153	42	8 (19.7)	0.4 (0.2, 1.1)	111	21 (18.9)	1.1 (0.6, 2.0)
No. of regular sexual partners							
≤1	182 (74.0)	31	7 (22.6)	1.0	151	33 (21.8)	1.0
>1	64 (26.0)	17	4 (23.5)	1.1 (0.3, 4.3)	47	6 (12.8)	0.5 (0.2, 1.3)
Condom use with sexual clients^a^							
≥Most of the time	255 (73.3)	56	19 (33.9)	1.0	199	34 (17.1)	1.0
<Most of the time	92 (26.5)	28	4 (14.3)	0.3 (0.1, 1.1)	64	14 (21.9)	1.4 (0.7, 2.7)
Condom use with regular sexual partners^b^							
≥Most of the time	62 (25.2)	23	6 (26.1)	1.0	39	5 (12.8)	1.0
<Most of the time	184 (74.8)	25	5 (20.0)	0.7 (0.2, 2.7)	159	34 (21.4)	1.8 (0.7, 5.1)
CD4 median (range), cells/mm^3 a^	910 (82–3567)	478 (152–1391)		1.2 (0.9, 1.5)^c^	1072 (82–3567)		1.0 (0.9, 1.1)^c^
*Chlamydia trachomatis*							
Negative	332 (96.2)	83	23 (27.7)		249	43 (17.3)	1.0
Positive	13 (3.8)	0	0 (0.0)	–	13	5 (38.5)	3.0 (0.9, 9.6)
*Neisseria gonorrhoeae*							
Negative	338 (97.7)	81	23 (28.4)		257	47 (18.3)	1.0
Positive	8 (2.3)	2	0 (0.0)	–	6	1 (16.7)	0.9 (0.1, 7.8)
*Trichomonas vaginalis*							
Negative	321 (92.8)	73	20 (27.4)	1.0	248	45 (18.2)	1.0
Positive	25 (7.2)	10	3 (30.0)	1.1 (0.3, 4.9)	15	3 (20.0)	2.7 (0.6, 11.7)
*Mycoplasma genitalium*							
Negative	301 (87.0)	71	18 (25.4)	1.0	230	42 (18.3)	1.0
Positive	45 (13.0)	12	5 (41.7)	2.1 (0.6, 7.5)	33	6 (18.2)	1.0 (0.4, 2.6)

CI: confidence intervals; HPV: Human papillomavirus; hrHPV: high-risk HPV; lrHPV: low-risk HPV; OR: odds ratios.

^a^Numbers do not add up to total due to missing values: education (n = 2), condom use with sexual clients (n = 1), CD4 (n = 81), *Chlamydia trachomatis* (n = 3), *Neisseria gonorrhoeae* (n = 2), *Trichomonas vaginalis* (n = 2), *Mycoplasma genitalium* (n = 2).

^b^Among women with regular sexual partners only (n = 246).

^c^Odds ratio for CD4 cell count is per 100 cell count change.

### Baseline HPV prevalence and cervical dysplasia

At baseline, 82 (23.6%) women were positive for any type of HPV (71 women had hrHPV types and 11 had lrHPV types) (Table 2). Overall HPV prevalence was notably higher in HIV-positive women (32.1%) compared to HIV-negative women (20.8%, p = 0.03). The prevalence of hrHPV types was 27.4% in HIV-positive women and 18.2% in HIV-negative women (p = 0.07). For cytology findings, 282 (81.0%) of FSWs were cytology normal, 14 (4.0%) had ASCUS/AGUS, 37 (10.6%) had LSIL, and 15 (4.3%) had HSIL/SCC. HIV-positive participants had a higher prevalence of advanced lesions (HSIL/SCC) (13.1%) than HIV-negative participants (1.5%, p < 0.01).

**Table 2.** Baseline prevalence of HPV DNA infection and cervical dysplasia stratified by baseline HIV status among 348 female sex workers in Nairobi, Kenya.

Characteristic	Totaln (%)(n = 348)	HIV-positiven (%)(n = 84)	HIV-negativen (%)(n = 264)	p-value^a^
HPV DNA^b^				
Any HPV	82 (23.6)	27 (32.1)	55 (20.8)	0.03
hrHPV	71 (20.4)	23 (27.4)	48 (18.2)	0.07
lrHPV	11 (3.2)	4 (4.8)	7 (2.7)	0.31
Cytology				
Normal	282 (81.0)	53 (63.1)	229 (86.7)	<0.01
ASCUS/AGUS	14 (4.0)	4 (4.8)	10 (3.8)	0.75
LSIL	37 (10.6)	16 (19.1)	21 (8.0)	<0.01
HSIL/SCC	15 (4.3)	11 (13.1)	4 (1.5)	<0.01
Histology				
Negative^c^	329 (94.5)	70 (83.3)	259 (98.1)	<0.01
CIN2+	19 (5.5)	14 (16.7)	5 (1.9)	<0.01

AGUS: atypical glandular cells of undetermined significance; ASCUS: atypical squamous cells of undetermined significance; CIN: cervical intraepithelial neoplasia; CIN2+: high grade CIN; HPV DNA: human papillomavirus deoxyribonucleic acid; hrHPV: high-risk HPV; HSIL: high-grade squamous intraepithelial lesion; lrHPV: low-risk HPV; LSIL: low-grade squamous intraepithelial lesion; SCC: squamous cell carcinoma.

^a^Based on Fisher’s exact test comparing HIV-positive to HIV-negative women α = 0.05.

^b^High-risk types: HPV16, 18, 31, 33, 35, 39, 45, 51, 52, 56, 58, 66, and 68/73; low-risk types: HPV6, 11, 44, 53, 54, 70, and 74.

^c^Histology-negative women included women with normal cytology who were not referred for colposcopy.

The prevalence of histologically-confirmed CIN2 or greater (CIN2+) was 5.5% overall, with a significantly higher prevalence among HIV-positive (16.7%) compared to HIV-negative women (1.9%, p < 0.01). Most HSIL/SCC were HPV positive (13/15, 86.7%), and of these, 7/13 (53.8%) were HPV52 positive (including n = 5 single HPV52 infections, n = 1 HPV52/31/54, n = 1 HPV52/33/54), two were HPV35 positive (single type infection), and there was one positive each for HPV16 (single type infection), HPV31 (within multiple infections: 31/52/54), HPV33 (33/52/54), HPV45 (45/56/74), HPV54 (31/52/54; 33/52/54), HPV66 (single type infection) and HPV70 (single type infection). The nine HPV vaccine-associated types included in the nonavalent vaccine (16, 18, 31, 33, 45, 52, 58, 6, and 11) made up 69.2% of the HPV positive HSIL/ICC cases.

Odds ratios for hrHPV positivity by baseline characteristics were not statistically significant (Table 1).

### HPV infection types

**Figure 1.**
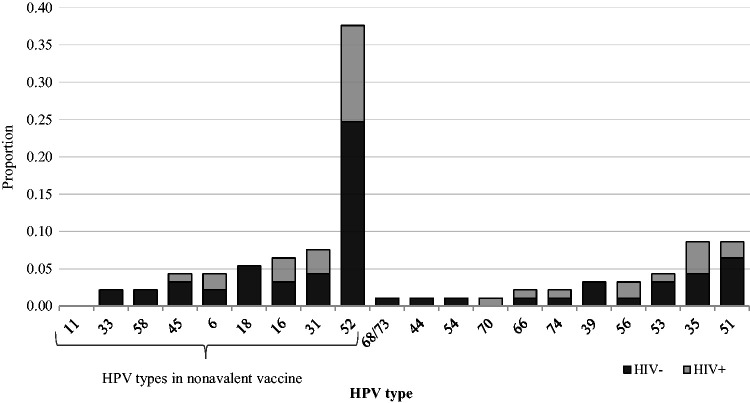
Baseline distribution of HPV types in a cohort of 348 FSWs in Nairobi, Kenya. HPV: human papillomavirus.

Single type HPV infections were detected in 68 (82.9%) women, 11 women (13.4%) had two HPV types, and 3 (3.7%) were positive for three types. The most common HPV type at baseline was HPV52 (10.1%), followed by HPV35 (2.3%), and HPV51 (2.3%) for single or multiple HPV type infections (Figure 1). HPV52 was the most predominant type in both HIV-positive (14.3%; n = 12/84) and HIV-negative (8.7%; n = 23/264) participants at baseline. HPV16 and HPV18 had a prevalence of 1.7 and 2.0%, respectively.

### Incident HPV infections

**Figure 2.**
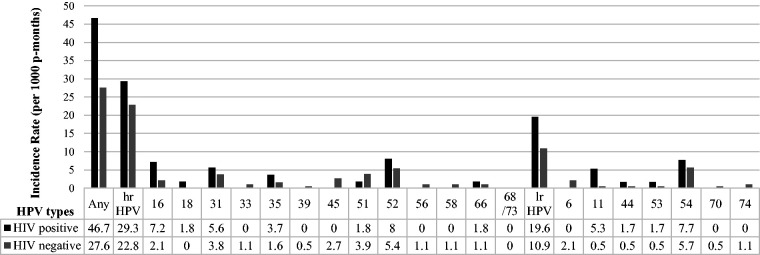
IRs of any HPV, hrHPV and lrHPV, among 160 FSWs with 12 or more months of follow-up, Nairobi, Kenya. hrHPV: high-risk HPV; lrHPV: low-risk HPV.

Of the 207 women with 12 months or more of follow-up (median 368 days, range 327–501), 47 women had HPV infections at baseline (22.7%). Of the 160 HPV-negative women at baseline, 50 (31.3%) developed incident HPV infections over study follow-up (Table 3). The majority (42/50, 84.0%) of incident infections were hrHPV. The IRs were 31.4 (95% CI: 23.8–41.5) for any HPV, 24.2 (95% CI: 17.9–32.8) for hrHPV types, and 12.8 (95% CI: 8.7–18.6) for lrHPV types. The IR was highest for HPV52 (IR = 6.0, 95% CI: 3.5–10.3). Stratified by HIV status (Figure 2), IR for overall HPV was higher in HIV-positive women (IR: 46.7, 95% CI: 28.2–77.5) than HIV-negative women (27.6, 95% CI: 19.8–38.4). Similarly, hrHPV IR was higher in HIV-positive (IR: 29.3, 95% CI: 16.2–52.9) than in HIV-negative women (IR: 22.8, 95% CI: 16.0–32.4). In HIV-positive women, the most common hrHPVs were HPV52, followed by HPV16 and HPV31. In HIV-negative women, hrHPV52 was also the most common, followed by HPV51 and HPV31.

**Table 3.** Incidence of any HPV, high-risk HPV and low-risk HPV with 95% CI among 160 female sex workers with 12 or more months of study follow-up, Nairobi, Kenya.

HPV type	Incident HPV infection	Subjects at risk (n)	Person-months of follow-up	IR^a^	(95% CI)^a^
Any HPV	50	160	1590	31.4	23.8, 41.5
High-risk HPV^b^	42	167	1734	24.2	17.9, 32.8
16	8	204	2418	3.3	1.6, 6.6
18	1	204	2439	0.4	0.1, 2.9
31	10	202	2352	4.3	2.3, 7.9
33	2	205	2448	0.8	0.2, 3.3
35	5	203	2421	2.1	0.8, 4.9
39	1	205	2457	0.4	0.1, 2.9
45	5	205	2439	2.1	0.8, 4.9
51	8	201	2358	3.4	1.7, 6.8
52	13	189	2175	6.0	3.5, 10.3
56	2	205	2454	0.8	0.2, 3.3
58	2	205	2448	0.8	0.2, 3.3
66	3	206	2445	1.2	0.4, 3.8
68/73	0	206	2484	0.0	0.0, 0.0
Low-risk HPV	27	191	2115	12.8	8.7, 18.6
6	4	205	2430	1.6	0.6, 4.4
11	4	207	2460	1.6	0.6, 4.3
44	2	206	2460	0.8	0.2, 3.3
53	2	206	2460	0.8	0.2, 3.3
54	14	199	2286	6.1	3.6, 10.3
70	1	206	2469	0.4	0.1, 2.9
74	2	204	2442	0.8	0.2, 3.3

CI: confidence interval; HPV: human papillomavirus; IR: incidence rate.

^a^Results based on using midpoint for incidence when HPV occurs within a six-month period.

^b^Infections with multiple types were considered high-risk if at least one infection was of high-risk type.

## Discussion

In this cohort of 348 FSWs in Kenya, HIV-positive women had a higher prevalence of overall HPV infection, hrHPV infection, and of cervical abnormalities as compared to HIV-negative women. In the longitudinal analysis, we also observed a notably higher HPV and hrHPV incidence in HIV-positive than HIV-negative women. HPV52 was the most commonly detected genotype at baseline and during follow-up among both HIV-positive and HIV-negative women.

The baseline prevalence of HPV in this cohort was 23.6%, with notably high detection in HIV-positive (32.1%) as compared to HIV-negative women (20.8%). hrHPV prevalence was 20.4% with higher detection in HIV-positive (27.4%) compared to HIV-negative (18.2%) women. Other studies in sub-Saharan Africa have observed similar differences in HPV prevalence by HIV status, with study-specific prevalence ranging widely from 52 to 72% in HIV-positive women and 21–47% in HIV-negative women.^12,18,24–26^ A study of FSWs in Rwanda (median age 25 years) reported prevalence of 72% in HIV-positive and 42% in HIV-negative women.^18^ hrHPV prevalence was also higher in HIV-positive (50.8%) than in HIV-negative women (31.8%). In South Africa, HPV prevalence in a general population was also higher in HIV-positive women (52.4%) than HIV-negative women (20.8%), with highest prevalence among younger women 17–19 years old.^12^ In a study of Kenyan HIV-positive women, De Vuyst et al.^24^ reported a baseline prevalence of 69% for all HPV DNA types and 52.6% for hrHPV DNA types. Separately, another study of 220 women in Kenya^25^ found an overall hrHPV prevalence of 37.7% (47% versus 27.6% in HIV-positive versus HIV-negative women). A meta-analysis of Kenyan studies in HIV-positive women (n = 13) supports these findings, reporting a pooled prevalence of 64%, with higher hrHPV pooled prevalence among FSWs compared to non-FSWs.^26^ The variable prevalence of HPV infections could be due to study-specific characteristics such as differences in age, geographical location, methods of HPV detection, and sociodemographic risk factors across the published studies. For instance, the study in Kenyan HIV-positive women^24^ included only HIV-positive women whereas our study had both HIV-positive and HIV-negative women (24% baseline HIV-positive prevalence). Further, the levels of immunosuppression in participants were different across studies. HIV-infected women with low CD4 cell count are at elevated risk for reactivation of HPV.^26^ Only 28% of participants from De Vuyst et al. had CD4 cell count >500 cells/mm^3^ whereas in our study, HIV-infected participants were generally not severely immunocompromised, with 40/81 (49%) with CD4 cell count >500 cells/mm^3^. It is also possible that unmeasured confounders in our study, such as length and use of antiretroviral therapy, could help explain some of the observed differences.

The HPV-positive prevalence observed in our study was lower than would be expected based on published literature. Factors such as sample preparation and how HPV laboratory assays are run may affect HPV detection across studies. An earlier study conducted in our cohort, using an Aptima assay for HPV mRNA detection reported an hrHPV prevalence (29%).^27^ Our study used a conservative approach, geared to specificity, in the detection of HPV types. Samples were initially tested for HPV positivity using the DEIA assay which can identify 44 known HPV types but does not provide typing information. The SPF10-LiPA^25^ assay was then used to determine specific types. The samples that were positive for HPV DNA by the DEIA assay but negative for the typing assay were excluded from HPV typing analyses. Thus, the lower rate of HPV reported in this study may be a result of the conservative approach geared toward the identification of specific HPV types.

HIV infection is a risk factor for HPV infection and the development of HPV-associated cervical lesions. HIV infection and HIV-associated immunosuppression may increase a woman’s susceptibility to HPV infection and alter the natural history of a pre-existing HPV infection.^4,5^ HIV-related immunosuppression is associated with increased HPV persistence and progression in the severity of cervical precursor lesions.^4^ Overall HSIL prevalence was 4.3%, with significantly higher prevalence in HIV-positive (13.1%) compared to HIV-negative (1.5%) women. Similarly, the CIN2+ prevalence (5.5% overall) was significantly higher in HIV-positive (16.7%) than in HIV-negative women (1.9%). Our findings are consistent with other studies,^12,16^ including a study of 9421 women drawn from the general population in Cape Town, South Africa,^12^ which found a significantly higher prevalence of CIN2+ among HIV-positive (9.2%) than HIV-negative women (2.7%). In a cohort of Kenyan FSWs where a third of the women were HIV-positive, the prevalence of HSIL was 4.8% in HIV-positive and 2.4% in HIV-negative women.^16^

In our study, 13/15 of the HSIL/SCC were HPV positive, supporting the well-established etiological role of HPV in cervical carcinogenesis. The most commonly detected HPV type in both HIV-positive and HIV-negative women was HPV52, with 10.1% prevalence at baseline, and an IR of 6.0 per 1000 person-months (95% CI: 3.5–10.3). Some^18,26,28^ although not all^7^ studies in sub-Saharan Africa have reported HPV52 as a commonly detected type in population-representative samples. A meta-analysis of HIV-positive women in Kenya found HPV52 was most prevalent (pooled estimate 26%) in women with normal cytology (n = 5 studies). However, among women with abnormal cytology, the most prevalent types were HPV16 followed by HPV35 and HPV52 in HSIL and HPV16 followed by HPV18 in ICC.^26^ In a Rwandese study of FSWs, HPV52 was most commonly detected in prevalent and incident analyses in HIV-positive women, and third most common among HIV-negative women.^18^ Evidence suggests that HIV-infected women may have a different HPV type distribution compared to the general population, with a relatively high prevalence of types other than 16 and 18.^29^

To our knowledge, the present study is among the first prospective studies that have used the SPF10-LiPA25 assay^30^ in sub-Saharan Africa; one other study used the SPF10-LiPA25 assay to examine HPV clearance in Nigerian women.^28^ A possible caveat of the SPF10-LiPA25 assay is that it does not differentiate between HPV68, an hrHPV type, from HPV73, a lrHPV type. All HPV68/73 were scored as hrHPV, which may lead to some misclassification, particularly if HPV73 were more prevalent than HPV68. In our study, the baseline prevalence of HPV68/73 was 0.28% and no incident HPV68/73 infections were detected in the longitudinal analysis. It is therefore unlikely that the HPV68/73 classification substantially influenced our results.

Among study strengths, first, the cervical smears were independently read by two cytopathologists, and any discrepant cases were graded based on a consensus of the two to ensure accurate cytologic diagnoses. Second, the prospective study design allowed the identification of incident HPV infections over the study follow-up. Additionally, we collected data on participants’ HIV status in order to observe notable differences in infection and disease prevalence stratified by HIV status in this FSW population. Our study has some limitations. Our study lacked complete follow-up on 141 participants, which could have led to selection bias. We conducted analyses comparing participants lost to follow-up to those with complete one-year follow-up data. We found similar baseline distribution of behavioral characteristics and prevalence of cervical disease across the two groups (data not shown). As such, loss to follow-up likely had minimal impact in our incidence analysis findings and interpretation.

Our findings were drawn from FSWs who may differ from the general population and this should be considered in interpreting the results. FSWs may have a higher risk of acquiring STIs due to their higher number of sexual partners and greater frequency of sexual encounters. In our FSW study population, the prevalence of STIs was relatively low (2.3% for GC, 3.8% for CT, 7.2% for TV, and 13.0% for MG), as compared to previous studies of FSWs in Africa. For example, among Rwandese HIV-negative FSWs, the prevalence was GC = 10%, CT = 5%, TV = 17%.^18^ In a study of FSWs in western Kenya, TV prevalence was 31.4%.^31^ The majority of women enrolling in our ancillary study of HPV were part of the Korogocho cohort study of FSWs originally established to better understand the immunogenesis of CT infections.^32^ As such, a large proportion of our population of FSWs reported using condoms with sexual clients ‘always or most of the time’ (73.3%) and this may have contributed to the lower STI prevalence. In contrast, 21.7% of women reported ‘regular condom use’ in the Kenyan FSW study,^31^ and 24% reported ‘always used condom in past month’ in the Rwandese FSW study.^18^

The higher prevalence and incidence of HPV, and associated high-grade cervical disease observed in HIV-positive women indicates that HIV-positive women should be a priority for public health interventions to reduce ICC morbidity and mortality. Our study was conducted among FSWs in a resource-limited setting, with a notable proportion (24%) having HIV infection, and this should be taken into account when considering extrapolation of these findings to populations that might be at lower risk for STIs. It is notable that despite years of sex work, 160/207 (77.3%) women were HPV negative at baseline, and 50 (31.3%) developed incident HPV infections over study follow-up. Most of these infections were hrHPV with high rates of non-16/18 hrHPV exposures. Prevention strategies, including HPV vaccination, the screening and treatment of cervical precancerous lesions, are critically needed globally to prevent ICC in both HIV-positive and HIV-negative women.
